# Noise Decomposition Principle in a Coherent Feed-Forward Transcriptional Regulatory Loop

**DOI:** 10.3389/fphys.2016.00600

**Published:** 2016-11-30

**Authors:** Rong Gui, Quan Liu, Yuangen Yao, Haiyou Deng, Chengzhang Ma, Ya Jia, Ming Yi

**Affiliations:** ^1^Department of Physics and Institute of Biophysics, Huazhong Normal UniversityWuhan, China; ^2^Department of Physics, College of Science, Huazhong Agricultural UniversityWuhan, China; ^3^Institute of Applied Physics, College of Science, Huazhong Agricultural UniversityWuhan, China

**Keywords:** coherent feed-forward loop, noise propagation, noise decomposition, linear noise approximation, expression noise, 87.18.Tt, 87.16.dj, 87.17.Aa

## Abstract

Coherent feed-forward loops exist extensively in realistic biological regulatory systems, and are common signaling motifs. Here, we study the characteristics and the propagation mechanism of the output noise in a coherent feed-forward transcriptional regulatory loop that can be divided into a main road and branch. Using the linear noise approximation, we derive analytical formulae for the total noise of the full loop, the noise of the branch, and the noise of the main road, which are verified by the Gillespie algorithm. Importantly, we find that (i) compared with the branch motif or the main road motif, the full motif can effectively attenuate the output noise level; (ii) there is a transition point of system state such that the noise of the main road is dominated when the underlying system is below this point, whereas the noise of the branch is dominated when the system is beyond the point. The entire analysis reveals the mechanism of how the noise is generated and propagated in a simple yet representative signaling module.

## Introduction

The biological world is filled with interaction of deterministic laws and randomness (Monod, 1972). Fluctuation and noise have penetrated into every level of biology, from the most essential molecular, sub-cellular processes to the kinetics of tissues, organs, organisms, and populations (Tsimring, 2014). In a biochemical reaction system, it is known that the external noise and the internal noise are both unavoidable (Harada et al., 1999; Hasty et al., 2000; Swain et al., 2002). The internal noise (referred to expression noise here) originates from random fluctuations of stochastic chemical reaction events (McAdams and Arkin, 1997; Arkin et al., 1998; Barkai and Leibler, 2000; Rao et al., 2002) in finite-size biochemical systems, which can lead to cell-to-cell variability. Increasing evidence suggests that this expression noise has significant impacts on many cellular processes.

A prominent feature of gene transcription regulatory networks is the presence of a large number of motifs, i.e., patterns of interconnection. These motifs include the auto-regulation loop, feedback loop, feed-forward loop and so on. An important task in the post-genome era is to understand how these different regulation mechanisms of expression noise affect the functioning of cells and how they contribute to cell-to-cell variability. Our ultimate purpose is to understand how the characteristics of noise in the complex networks can be derived from the properties of modules that are used to compose these networks.

It has been recognized that feedback loops play significant roles in a variety of biological processes, such as calcium signaling (Berridge, 2001; Lewis, 2001), p53 regulation (Harris and Levine, 2005), galactose regulation (Acar et al., 2005), cell cycle (Morgan, 2006; Yang et al., 2013; Liu et al., 2015), and cell fate decision in budding yeast (Li et al., 2014, 2015). Some studies suggested that negative feedbacks typically attenuated noise and positive feedbacks tended to amplify noise (Becskei and Serrano, 2000; Austin et al., 2006; Alon, 2007); however, there were other studies revealing that positive feedbacks could attenuate noises and there were no strong correlations between the sign of feedbacks (negative or positive) and the noise attenuation properties (Hooshangi and Weiss, 2006; Hornung and Barkai, 2008).

It is well-known that the feed-forward loop is also a typical of biological motif. The feed-forward loop, a pattern of three genes, consists of two input transcription factors, one of which regulates the other, conjointly regulating a target gene. Each of the interactions of three genes in the feed-forward loop can be activation or inhibition so that the feed-forward loop has eight possible structural types. Among them, the coherent feed-forward loop appears with the highest frequency in the organism (Mangan and Alon, 2003). The structures, functions, as well as noise characteristics of feed-forward loop have received increasing attention over the last decade (Mangan and Alon, 2003; Mangan et al., 2003, 2006; Ghosh et al., 2005; Dekel et al., 2005; Kalir et al., 2005; Prill et al., 2005; Wall et al., 2005; Alon, 2006, 2007; Kaplan et al., 2008; Kim et al., 2008; Goentoro et al., 2009; Guo and Li, 2009; Macía et al., 2009; Kittisopikul and Suel, 2010; Sontag, 2010).

Few studies, however, focused attentions on the effect of feed-forward on expression noise in biochemical systems. The current study has ever studied the mechanisms of feed-forward regulation in cell fate decisions in budding yeast (Li et al., 2015). As is shown in (Mangan and Alon, 2003), the authors have found that the coherent feed-forward loop rejects transient input pulses and responds only to persistent stimuli. The feed-forward loop attains their steady states if the stimuli act over a sufficiently long time interval. In the paper (Ghosh et al., 2005), the noise characteristics of coherent and incoherent feed-forward loop in the steady state have been studied using the Langevin formalism as well as a numerical simulation. However, it is still unclear how expression noise is associated with the feed-forward structure. Furthermore, a challenging task is to trace the sources of expression noise and elucidate their roles in the feed-forward-mediated pathway. In addition, how noise is propagated in the feed-forward loop has not been fully solved.

To address the above questions, fluctuation and noise propagation in the coherent feed-forward transcriptional regulatory loop are investigated in this paper. Our motivation is to clarify the potential relationships between network structure, noise characteristics, and biological function. The main contribution of our study is that we decompose the expression noise of each element in the coherent feed-forward loop into different noise sources (denoted as fine structure here). We believe that our study presents a possible understanding for why has the biological system evolved into a coherent feed-forward regulatory mechanism. This paper is organized as follows. In Section Mathematical modeling and analytical noise, the mathematical models of coherent feed-forward loop, including its main road and branch subsystems, are presented first. Then the related theoretical methods are introduced to calculate variances and normalized variations of each expression production in these motifs. Further, in Section Results, we analyse the fluctuation and noise propagation in the coherent feed-forward loop and its subsystems. Finally, the conclusions and discussions are given in Section Conclusion.

## Mathematical modeling and analytical noise

**Figure 1:**
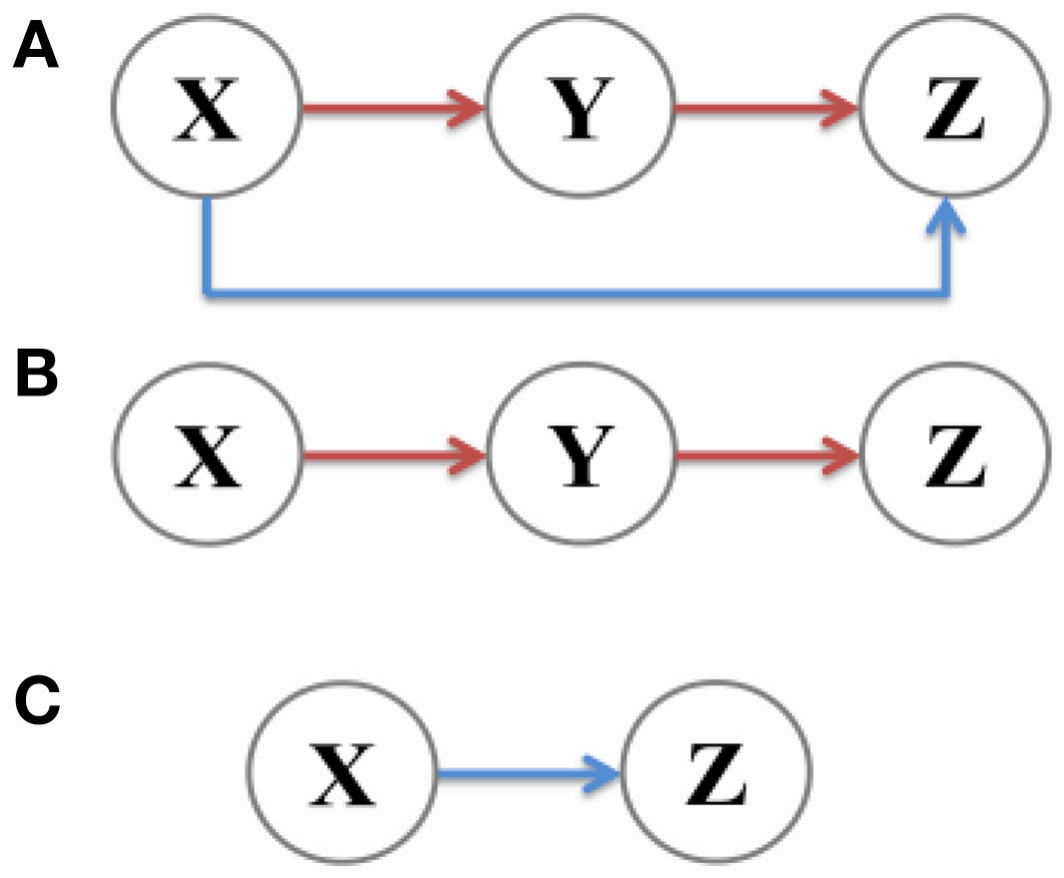
**Signal transmission in different gene motifs, including (A)** coherent feed-forward loop, **(B)** main road, i.e., two-step cascade, and **(C)** branch, i.e., one-step cascade.

A coherent feed-forward loop is composed of three components: Two transcription factors X and Y, where the former regulates the latter, and a target gene Z, where X and Y both bind the regulatory region of Z and jointly modulate the transcription rate (Figure 1A). In order to fully investigate noise characteristics in the coherent feed-forward loop, two subsystems, the main road (Figure 1B) and branch (Figure 1C), are chosen for comparison. The main road is a two-step cascade, through which transcription factor X regulates the expression of transcription factor Y and Y regulates Z. The branch is a one-step cascade, namely transcription factor X regulate target gene Z directly, which acts as a regulatory pathway. Therefore, we will explore how the intrinsic noise is propagated in the coherent feed-forward loop, also including the one-step cascade (i.e., branch) and the two-step cascade (i.e., main road). Below, X means the upstream factor, Y represents the intermediate component, and Z is the downstream element. In our research, the analytical formulas of noise are derived by using the linear noise approximation (Kampen, 2007). The numerical results with Gillespie method (Gillespie, 1977) are used to compare with the analytic results.

### Deterministic model and steady state

Based on the biochemical reaction rules, the mathematical models of these corresponding gene regulation motifs are built by virtue of ordinary differential equations. Two conditions are considered, respectively.

**Figure 2:**
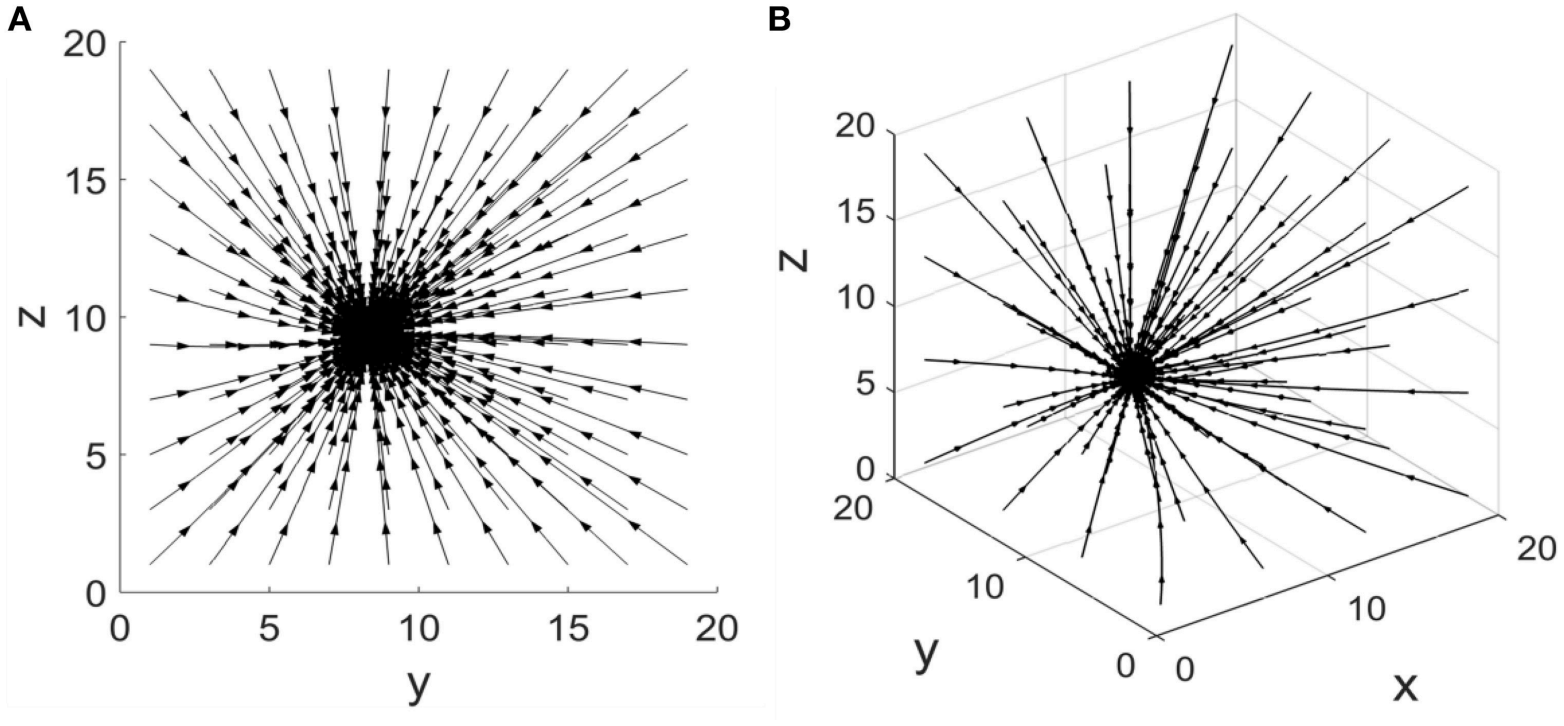
**Phase portrait of the coherent feed-forward loop**. **(A)** Treating the concentration of X as an adjustable parameter.**(B)** Treating the concentration of X as a variable.

- Treating the level of X as an adjustable parameter.
  First, the expression process of X is neglected and the concentration of X is regarded as the control parameter. Hence, the model of coherent feed-forward loop has only two variables. The deterministic dynamics can be described by the following equations:
  (1) $$\begin{array}{l} \frac{dy}{dt}=\alpha_{y}\omega_{1}\frac{x}{K+x}-\delta_{y}y, \end{array}$$
  (2) $$\begin{array}{l} \frac{dz}{dt}=\alpha_{z}\frac{\omega_{2}x+y}{K+\omega_{2}x+y}-\delta_{z}z, \end{array}$$
  where *x, y*, and *z* are the concentration variables for X,Y, and Z, α_*y*_ and α_*z*_ are the maximum level of activated protein production for Y and Z, which are set to ten, respectively. *K* is the corresponding Hill constant, which is set to unity. The dilution and degradation rate of Y and Z are δ_*y*_ and δ_*z*_, which are set to unity, respectively. The model of the branch is defined by ω_1_ = 0, ω_2_ = 1, the main road by ω_1_ = 1 and ω_2_ = 0, and the coherent feed-forward loop by ω_1_ = 1 and ω_2_ = 1.
  For equations (1) and (2), there exists an attracting fixed point $\left(\alpha_{y}\omega_{1}\frac{x}{\delta_{y}\left(K+x\right)},\alpha_{z}\frac{\omega_{2}x+y}{\delta_{z}\left(K+\omega_{2}x+y\right)}\right).$ The corresponding phase portrait of this coherent feed-forward loop is illustrated in Figure 2A when the concentration of X is five. It shows that the system converges to a final steady state when the system starts from different initial states, i.e., the attractor of the system. Furthermore, from the phase plane, it is observed that each trajectory from different initial states is nearly close to a straight line, only a small number of trajectories are a little curved. In the case of other parameters, the phase diagram is qualitatively invariant.
- Treating the level of X as a variable.
  Then, we consider the birth and death processes of X. Therefore, the model of coherent feed-forward loop has three variables whose dynamics can be described by the following equations:
  (3) $$\begin{array}{l} \frac{dx}{dt}=\alpha_{x}-\delta_{x}x, \end{array}$$
  (4) $$\begin{array}{l} \frac{dy}{dt}=\frac{\alpha_{y}\omega_{1}x}{K+x}-\delta_{y}y, \end{array}$$
  (5) $$\begin{array}{l} \frac{dz}{dt}=\frac{\alpha_{z}\left(\omega_{2}x+y\right)}{\left(K+\omega_{2}x+y\right)}-\delta_{z}z. \end{array}$$
  Similarly, the branch is defined by ω_1_ = 0, ω_2_ = 1, the main road by ω_1_ = 1 and ω_2_ = 0, and the coherent feed-forward loop by ω_1_ = 1and ω_2_ = 1. We will consider α_*x*_ as a control parameter under this condition. α_*y*_ = α_*z*_ = 10, δ_*x*_ = δ_*y*_ = δ_*z*_ = 1.

Equations (3–5) have an attracting fixed point $\left(\frac{\alpha_{x}}{\delta_{x}},\alpha_{y}\omega_{1}\frac{x}{\delta_{y}\left(K+x\right)},\alpha_{z}\frac{\omega_{2}x+y}{\delta_{z}\left(K+\omega_{2}x+y\right)}\right)$. Phase portrait of this coherent feed-forward loop is also presented in Figure 2B when the production rate α_*x*_ is five. The three-dimensional phase diagram (Figure 2B) is similar to the previous two dimensional phase diagram (Figure 2A). Starting some different initial states, after almost straight trajectories, then the system finally reaches a stable state. For other parameters values, the phase diagram remains unchanged from the qualitative perspective.

### Stochastic model and derivation for the analytical formula of noise

**Figure 3:**
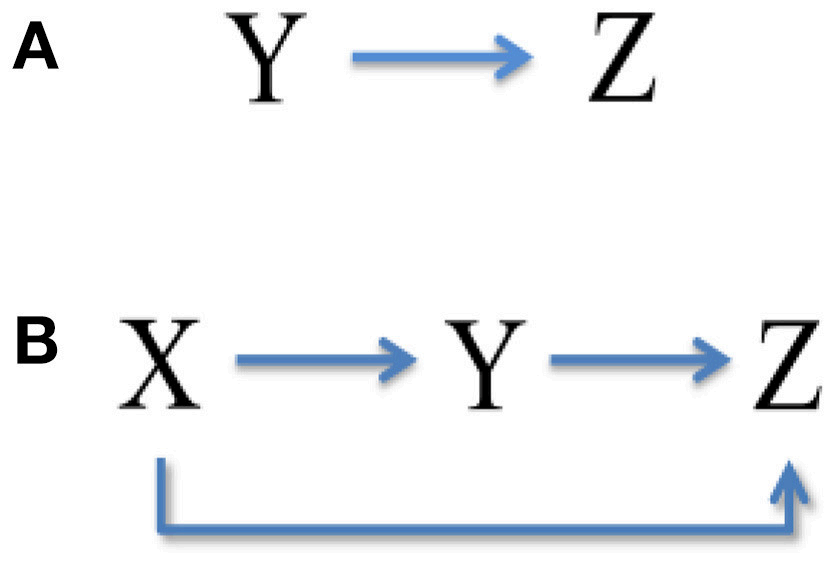
**Noise transmission in coherent feed-forward loop**. **(A)** Treating the concentration of X as an adjustable parameter. **(B)** Treating the concentration of X as a variable.

- Treating the level of X as an adjustable parameter.
  Under small molecular numbers, the stochastic noise can be described exactly with the master equations. A theoretical derivation about the noise expression is given below, starting from the master equations. The numbers of the expression productions can be expressed as *N*_*i*_, *i* ∈ {*x, y, z*}. Relatively, The numbers of the expression productions of Y and Z can be expressed as *N*_*y*_ = Ω*y*, *N*_*z*_ = Ω*z*, where Ω is defined as the size of system. Throughout the paper, Ω is set to 100.To characterize the noise, the linear noise approximation is adopted as below.
  The joint probability distribution *P*(*N*_*y*_, *N*_*z*_, *t*) of coupled ordinary differential equations Eqs. (1) and (2) obey the following master equation (Jia and Li, 1997; Kampen, 2007; Brett and Galla, 2013):
  (6) $$\begin{array}{l} \frac{dP}{dt}=\left[\begin{array}{l} \alpha_{y}\omega_{1}\left(E_{y}^{-1}-1\right)\frac{\Omega x}{K+x}+\left(E_{y}^{+1}-1\right)\delta_{y}N_{y}\\ \;+\;\alpha_{z}\frac{\left(\omega_{2}\Omega x+N_{y}\right)}{K+\omega_{2}x+\frac{N_{y}}{\Omega }}\left(E_{z}^{-1}-1\right)+\left(E_{z}^{+1}-1\right)\delta_{z}N_{z} \end{array}\right]P, \end{array}$$
  where the symbol *E* represents a step operator, which is defined as $E_{i}^{+1}f\left(N_{i}\right)=f\left(N_{i}+1\right),E_{i}^{-1}f\left(N_{i}\right)=f\left(N_{i}-1\right)$. The master equation cannot be solved exactly, so a systematic approximation method has been developed here. By using van Kampen's Ω -expansion method, the numbers of Y and Z is approximated by setting Ny=Ωy+Ω12ξy(t), Nz=Ωz+Ω12ξz(t). The joint probability distribution is written by $P\left(N_{1},N_{2},\ldots ,N_{R},t\right)=\Omega^{-R/2}\Pi \left(\xi_{1},\xi_{2},\ldots ,\xi_{R},t\right)$. Collecting the terms of Ω^1/2^ in the expansion of Equation (6) reproduces the concentration form of the macroscopic rate equation and the terms of Ω^0^ get a linear Fokker-Planck equation,
  (7) $$\begin{array}{l} \frac{\partial \Pi }{\partial t}=-\sum_{i,j}A_{ij}\partial_{i}\left(\xi_{j}\Pi \right)+\frac{1}{2}\sum_{i,j}B_{ij}\partial_{ij}\Pi . \end{array}$$
  *A* is the stationary Jacobian matrix of the deterministic equations. *B* is the stationary diffusion matrix.
  Thus,
  (8) $$\begin{array}{l} A=\left[\begin{array}{cc} -\delta_{y} & 0\\ \frac{\alpha_{z}K}{{\left(K+\omega_{2}x+y\right)}^{2}} & -\delta_{z} \end{array}\right], \end{array}$$
  (9) $$\begin{array}{l} B=\left[\begin{array}{cc} \frac{\alpha_{y}\omega_{1}x}{\left(K+x\right)}+\delta_{y}y & 0\\ 0 & \frac{\alpha_{z}\left(\omega_{2}x+y\right)}{\left(K+\omega_{2}x+y\right)}+\delta_{z}z \end{array}\right]. \end{array}$$
  The linear noise approximation is summarized by (Elf and Ehrenberg, 2003; Thomas et al., 2012)
  (10) $$\begin{array}{l} AC+CA^{T}+\Omega B=0, \end{array}$$
  where matrix *C* contains both the variances *C*_*yy*_, *C*_*zz*_,which characterizes the fluctuation in Y and Z. Substituting Equations (8) and (9) into Equation (10), we obtain
  (11) $$\begin{array}{l} C_{yy}=-\frac{\Omega B_{yy}}{2A_{yy}}, \end{array}$$
  (12) $$\begin{array}{l} C_{yz}=-\frac{C_{yy}A_{zy}}{A_{yy}+A_{zz}}=C_{zy}, \end{array}$$
  (13) $$\begin{array}{l} C_{zz}=-\frac{B_{zz}\Omega }{2A_{zz}}+\frac{A_{zy}}{A_{zz}}\frac{C_{yy}A_{zy}}{A_{yy}+A_{zz}}, \end{array}$$
  To quantify the noise propagation around the steady state, Equation (10) is normalized as
  (14) $$\begin{array}{lll} MV & + & V^{T}M^{T}+D=0, \end{array}$$
  $$\begin{array}{l} \text{with }V_{ik}=V_{ki}=\frac{C_{ik}}{<N_{i}><N_{k}>},\;M_{ik}=A_{ik}\frac{<N_{k}>}{<N_{i}>},\\ D_{ik}=\Omega \frac{B_{ik}}{<N_{i}><N_{k}>}. \end{array}$$
  To measure how the balance between production and elimination of *N*_*i*_ is affected by *N*_*k*_ (Paulsson, 2004, 2005; Pedraza and van Oudenaarden, 2005; Hornung and Barkai, 2008; Jia et al., 2009; Pei et al., 2015), *N*_*i*_ and *N*_*k*_ represent the numbers of the expression productions, respectively. *i, k* ∈ {*x, y, z*}. The logarithmic gain is defined by $H_{ik}=\frac{\partial ln\left(J_{i}^{-}/J_{i}^{+}\right)}{\partial ln\left(N_{k}\right)}$, where $J_{i}^{+}$ is the pure production rate and $J_{i}^{-}$ is the pure elimination rate of the expression production. *H*_*ik*_ represents a common method of the sensitivity of a response to changes in parameter *N*_*k*_, also known as logarithmic gain (Savageau, 1977), sensitivity amplification (Goldbeter and Koshland, 1982; Heinrich and Schuster, 1996), or susceptibility (Scott et al., 2006). Considering the constant death rates and the constant transition rates, we can get the logarithmic gains as follows
  (15) $$\begin{array}{l} H=\left[\begin{array}{cc} H_{yy} & H_{yz}\\ H_{zy} & H_{zz} \end{array}\right]=\left[\begin{array}{cc} 1 & 0\\ \frac{-yK}{\left(K+\omega_{2}x+y\right)\left(\omega_{2}x+y\right)} & 1 \end{array}\right]. \end{array}$$
  Under the steady state (i.e., $J_{i}^{-}=J_{i}^{+}=J_{i}$), the average lifetime τ_*i*_ is determined by the total rate of elimination, $\tau_{y}=y/J_{y}^{-}=1/\delta_{y},\tau_{z}=y/J_{z}^{-}=1/\delta_{z}\;\text{respectively}.\;$ Thus, the drift matrix A is represented by $\;A_{ik}=-\frac{<N_{i}>}{<N_{k}>}\frac{H_{ik}}{\tau_{i}}$.
  Then its normalized formation *M* is rewritten as
  (16) $$\begin{array}{l} M_{ik}=A_{ik}\frac{<N_{k}>}{<N_{i}>}=-\frac{H_{ik}}{\tau_{i}}. \end{array}$$
  In order to study noise propagation, we substitute Equation (15) into Equation (16), and then solve Equation (14) for the normalized variations *V*_*yy*_, and *V*_*zz*_. Hence we have
  (17) $$\begin{array}{l} V_{yy}=\frac{1}{\langle y\rangle H_{yy}}, \end{array}$$
  (18) $$\begin{array}{l} V_{zz}=\frac{1}{\langle z\rangle H_{zz}}+\frac{H_{zy}}{H_{zz}}\frac{H_{zy}}{H_{zz}}\frac{\frac{H_{zz}}{\tau_{z}}V_{yy}}{\left(\frac{H_{yy}}{\tau_{y}}+\frac{H_{zz}}{\tau_{z}}\right)}. \end{array}$$
  It is seen that the upstream X has no contribution to the total noise in downstream Z, while the intermediate Y transmits a part of expression noise to Z, as shown in Figure 3A. Noise from neighbor is called the one-step propagation noise.
  Equations (1) and (2) can be translated into the following set of birth-death processes:
  (19) $$\begin{array}{l} \emptyset \overset{k_{y}}{\to }y, \end{array}$$
  (20) $$\begin{array}{l} \emptyset \overset{k_{z}}{\to }z, \end{array}$$
  (21) $$\begin{array}{l} y\overset{\delta_{y}}{\to }\emptyset , \end{array}$$
  (22) $$\begin{array}{l} z\overset{\delta_{z}}{\to }\emptyset , \end{array}$$
  where Equations (19) and (20) describe the production of Y and Z,respectively. In Equations (19), $\;k_{y}=\alpha_{y}\omega_{1}\frac{x}{K+x}$, and in Equations (20), $k_{z}=\alpha_{z}\frac{\omega_{2}x+y}{K+\omega_{2}x+y}$. The degradations of Y and Z are described by Equations (21) and (22), respectively. Stochastic simulation of chemical reactions is performed using the Gillespie algorithm (Gillespie, 1977).
- Treating the level of X as a variable.
  The joint probability distribution *P*(*N*_*x*_, *N*_*y*_, *N*_*z*_, *t*) of coupled ordinary differential equations (Equations 3–5) obey the following master equation:
  (23) $$\begin{array}{l} \frac{dP}{dt}=\left[\begin{array}{c} \frac{\omega_{1}\alpha_{y}N_{x}}{K+\frac{N_{x}}{\Omega }}\left(E_{y}^{-1}-1\right)+\delta_{y}\left(E_{y}^{+1}-1\right)N_{y}\\ \;+\;\frac{\alpha_{z}\left(\omega_{2}N_{x}+N_{y}\right)}{\left(K+\frac{\omega_{2}N_{x}}{\Omega }+\frac{N_{y}}{\Omega }\right)}\left(E_{z}^{-1}-1\right)+\delta_{z}\left(E_{z}^{+1}-1\right)N_{z}\\ \;+\;\Omega \alpha_{x}\left(E_{x}^{-1}-1\right)+\delta_{x}\left(E_{x}^{+1}-1\right)N_{x} \end{array}\right]\;P, \end{array}$$
  Thus,
  (24) $$\begin{array}{l} A=\left[\begin{array}{ccc} -\delta_{x} & 0 & 0\\ \frac{\alpha_{y}\omega_{1}K}{{\left(K+x\right)}^{2}} & -\delta_{y} & 0\\ \frac{\alpha_{z}\omega_{2}K}{{\left(K+\omega_{2}x+y\right)}^{2}} & \frac{\alpha_{z}K}{{\left(K+\omega_{2}x+y\right)}^{2}} & -\delta_{z} \end{array}\right], \end{array}$$
  (25) $$\begin{array}{l} B=\left[\begin{array}{ccc} \alpha_{x}+\delta_{x}x & 0 & 0\\ 0 & \frac{\alpha_{y}\omega_{1}x}{K+x}+\delta_{y}y & 0\\ 0 & 0 & \frac{\alpha_{z}\left(\omega_{2}x+y\right)}{K+\omega_{2}x+y}+\delta_{z}z \end{array}\right].\; \end{array}$$
  The linear noise approximation is summarized by *AC* + *CA*^*T*^ + Ω*B* = 0. We get
  (26) $$\begin{array}{l} C_{xx}=-\frac{\Omega B_{xx}}{2A_{xx}}, \end{array}$$
  (27) $$\begin{array}{l} C_{yx}=-\frac{C_{xx}A_{yx}}{A_{xx}+A_{yy}}=C_{xy}, \end{array}$$
  (28) $$\begin{array}{l} C_{yy}=-\frac{\Omega B_{yy}}{2A_{yy}}-\frac{C_{xy}A_{yx}}{A_{yy}}, \end{array}$$
  (29) $$\begin{array}{l} C_{zx}=-\frac{A_{zx}C_{xx}+A_{zy}C_{yx}}{A_{xx}+A_{zz}}=C_{xz}, \end{array}$$
  (30) $$\begin{array}{l} C_{zy}=-\frac{A_{zx}C_{xy}+A_{zy}C_{yy}+C_{zx}A_{yx}}{A_{yy}+A_{zz}}=C_{yz}, \end{array}$$
  (31) $$\begin{array}{l} C_{zz}=-\frac{\Omega B_{zz}+2A_{zx}C_{xz}+2A_{zy}C_{yz}}{2A_{zz}}. \end{array}$$
  Taking into account the constants of both the self-proliferation rates and the death rates, the logarithmic gain *H*_*ik*_ can be obtained
  (32) $$\begin{array}{l} H=\left[\begin{array}{ccc} H_{xx} & H_{xy} & H_{xz}\\ H_{yx} & H_{yy} & H_{yz}\\ H_{zx} & H_{zy} & H_{zz} \end{array}\right]\\ =\left[\begin{array}{ccc} 1 & 0 & 0\\ \frac{-K}{K+x} & 1 & 0\\ -\frac{K\omega_{2}x}{\left(K+\omega_{2}x+y\right)\left(\omega_{2}x+y\right)} & \frac{-yK}{\left(K+\omega_{2}x+y\right)\left(\omega_{2}x+y\right)} & 1 \end{array}\right].\; \end{array}$$
  Under the steady state, the average lifetime τ_*i*_ is determined by the total rate of elimination, $\tau_{x}=x/J_{x}^{-}=1/\delta_{x},\tau_{y}=y/J_{y}^{-}=1/\delta_{y},\tau_{z}=y/J_{z}^{-}=1/\delta_{z}$ respectively. In order to study noise propagation, we substitute Equation (32) into Equation (16), and then solve Equation (14) for the normalized variations *V*_*xx*_, *V*_*yy*_, and *V*_*zz*_ here, and then we have
  (33) $$\begin{array}{l} V_{xx}\;=\frac{1}{\underset{Intrinsic\;noise}{\underset{︸}{H_{xx}\langle x\rangle }}}, \end{array}$$
  (34) $$\begin{array}{l} V_{yy}\;=\;\frac{1}{\underset{Intrinsic\;noise}{\underset{︸}{H_{yy}\langle y\rangle }}}+\underset{one-step\;propagation\;noise\;from\;X}{\underset{︸}{\frac{H_{yx}H_{yx}}{H_{yy}H_{yy}}\frac{\frac{H_{yy}}{\tau_{y}}}{\frac{H_{xx}}{\tau_{x}}+\frac{H_{yy}}{\tau_{y}}}V_{xx},}} \end{array}$$
  (35) $$\begin{array}{l} V_{zz}=\frac{1}{\underset{Intrinsic\;noise}{\underset{︸}{H_{zz}\langle z\rangle }}}+\underset{one-step\;propagation\;noise\;from\;X}{\underset{︸}{\frac{H_{zx}H_{zx}}{H_{zz}H_{zz}}\frac{\frac{H_{zz}}{\tau_{z}}}{\frac{H_{xx}}{\tau_{x}}+\frac{H_{zz}}{\tau_{z}}}V_{xx}}}\\ \;+\underset{one-step\;propagation\;noise\;from\;Y}{\underset{︸}{\frac{H_{zy}H_{zy}}{H_{zz}H_{zz}}\frac{\frac{H_{zz}}{\tau_{z}}}{\frac{H_{zz}}{\tau_{z}}+\frac{H_{yy}}{\tau_{y}}}V_{yy}}}\\ \;+\;V_{two-steps\;propagation\;noise\;from\;X}, \end{array}$$
  (36) $$\begin{array}{l} V_{\begin{array}{l} two-steps\;propagation\\ noise\;from\;X \end{array}}=\frac{H_{zy}}{H_{zz}}\frac{\frac{H_{zy}}{\tau_{z}}}{\frac{H_{zz}}{\tau_{z}}+\frac{H_{yy}}{\tau_{y}}}\frac{\frac{H_{yx}}{\tau_{y}}}{\frac{H_{zz}}{\tau_{z}}+\frac{H_{xx}}{\tau_{x}}}\frac{\frac{H_{yx}}{\tau_{y}}}{\frac{H_{xx}}{\tau_{x}}+\frac{H_{yy}}{\tau_{y}}}V_{xx}\\ \;-\;\frac{H_{zx}}{H_{zz}}\frac{\frac{H_{zy}}{\tau_{z}}}{\frac{H_{xx}}{\tau_{x}}+\frac{H_{zz}}{\tau_{z}}}\frac{\frac{H_{yx}}{\tau_{y}}}{\frac{H_{xx}}{\tau_{x}}+\frac{H_{yy}}{\tau_{y}}}V_{xx}\\ \;-\;\frac{H_{zy}}{H_{zz}}\frac{\frac{H_{yx}}{\tau_{y}}}{\frac{H_{zz}}{\tau_{z}}+\frac{H_{yy}}{\tau_{y}}}\frac{\frac{H_{zx}}{\tau_{z}}}{\frac{H_{zz}}{\tau_{z}}+\frac{H_{xx}}{\tau_{x}}}V_{xx}\\ \;-\;\frac{H_{zy}}{H_{zz}}\frac{\frac{H_{zx}}{\tau_{z}}}{\frac{H_{zz}}{\tau_{z}}+\frac{H_{yy}}{\tau_{y}}}\frac{\frac{H_{yx}}{\tau_{y}}}{\frac{H_{xx}}{\tau_{x}}+\frac{H_{yy}}{\tau_{y}}}V_{xx}. \end{array}$$

We can see that, accompanied by signal transduction in the network, the noise is also transmitted along these pathways. The upstream X has contributions to the total noise in Y and Z via one-step (branch road) and two-step (main road) propagation, respectively (see Figure 3B). Y also delivers a part of noise to Z by one-step propagation. It is noted that the analytic expression of total noise in Z seems to be complex. However, the third term *V*_*two*−*steps propagation noise from X*_ on the right describes the two-step propagation noise from X to Z.

## Results

Based on the theoretical formulas of variances and normalized variations obtained above, we can make a further analysis about how noise is transmitted in the coherent feed-forward loop. Some interesting results are observed.

### The total noise level of downstream component is always the smallest

In order to illustrate noise characteristic of different nodes in these small gene networks, as observed in Equations (18)and(35), we investigate the normalized variations of each element in a variety of situations, which can present a global feature.

**Figure 4:**
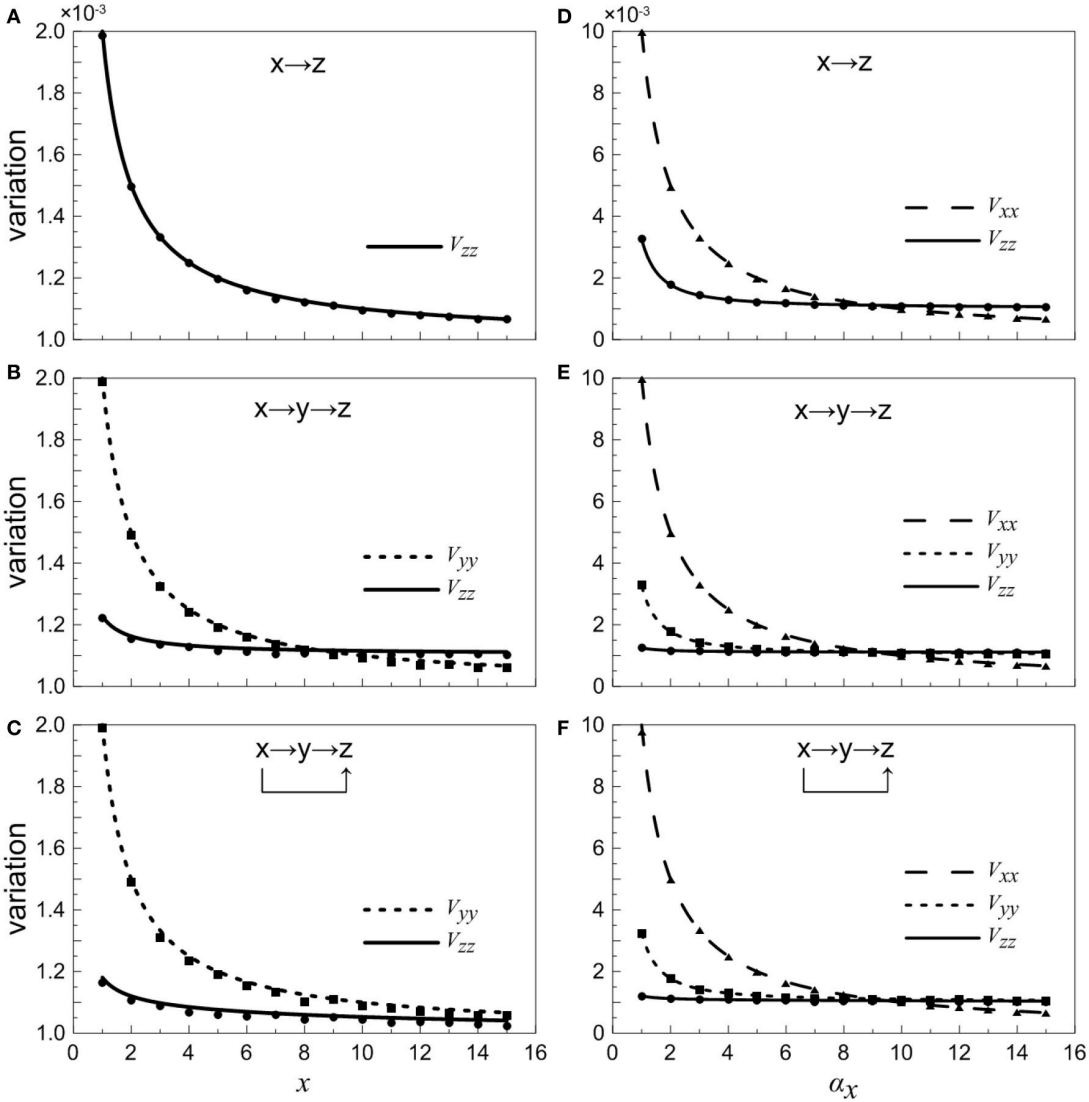
**Dependences of normalized variations of different genes on control parameters for different motifs**. **(A–C)** Treating the concentration of X as an adjustable parameter. **(D–F)** Treating the concentration of X as a variable. From top to bottom, the results for one-step cascade (branch), two-step cascade(main road) and coherent feed-forward loop are shown, respectively. Lines are theoretical predictions with Equations (17, 18) and (33–36), and solid markers are from simulations using the Gillespie method.

When the concentration of X is constant and regarded as a control parameter, the normalized variations *V*_*yy*_ and *V*_*zz*_ under different conditions are given in Figures 4A–C. It shows that with the increasing of *x*, the normalized noise levels in Y and Z both decrease. For the two-step cascade in the main road (Figure 4B), there is a critical value, below which the normalized variation of the Y is larger than that of Z. Beyond the critical point, the situation is slightly reversed, the noise of downstream component becomes larger than that of the upstream. However, for the coherent feed-forward loop, it can be found that the critical value almost disappears (Figure 4C). That is to say, no matter how much the value of *x* is, the normalized variation of Z is always smaller than that of Y. The result shows that, due to the addition of regulation branch, the noise of the downstream element becomes the smallest.

When we treat the production rate α_*x*_ as a control parameter, both X and Y have contributions to the total noise of Z. The noise curves of X are calculated and supplied in Figures 4D–F. The noise of X is the largest when α_*x*_ is less than certain threshold for all cases. When α_*x*_ increases to a certain value, the concentration of X increases, hence the intrinsic noise of X is reduced. It is illustrated that, in the region below a certain critical point, the total noise level of downstream component is the smallest compared with other upstream components. Basically, if the stochastic birth-death process of X is involved, the noise levels are enhanced, but the critical points are only modified slightly.

### Noise characteristics of gene Z

**Figure 5:**
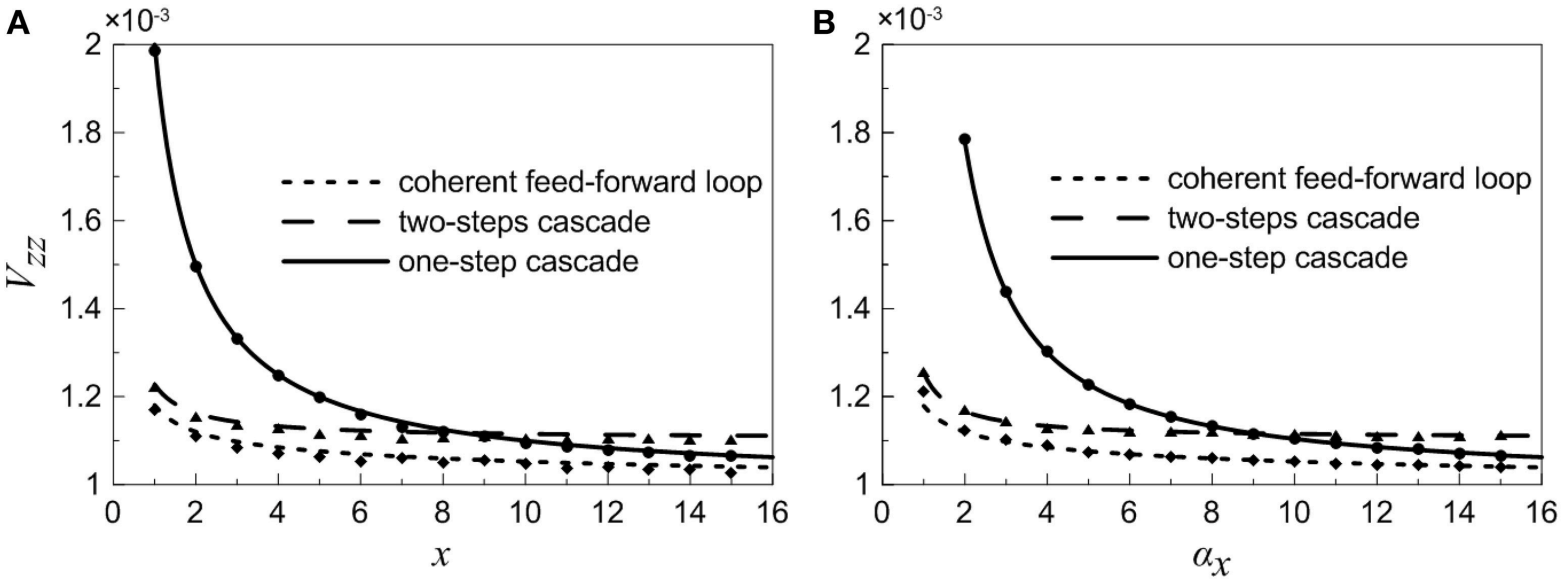
**Normalized variations of gene Z for different motifs**. **(A)** Treating the concentration of X as an adjustable parameter. **(B)** Treating the concentration of X as a variable. Lines are theoretical predictions with Equations (18), (35), and (36), and solid markers are from simulations using the Gillespie method.

Because the target gene Z is a downstream gene and can be considered as the system's output, we focus on the noise characteristics of gene Z. A comparison between the normalized variations *V*_*zz*_ for different motifs is plotted in Figure 5. It is observed that the noise curve of coherent feed-forward loop is the lowest especially when the control parameter α_*x*_ is small. Our theoretical findings reveal that the multi-step process is beneficial for signal transmission from X to Z, i.e., the coherent feed-forward loop can attenuate effectively the noise level of downstream gene. Therefore, living organisms could utilize feed forward for better survival in fluctuating environments.

For the one-step cascade, signal X can be propagated directly to Z, however, the noise level is always larger than that of the other motifs. In addition, the two noise curves of one-step cascade and two-step cascade intersect at a certain point.

### Noise decomposition

As is mentioned above, the total noise can be decomposed into a series of noise terms, and each noise term represents different sources. As observed in Equation (18), when X is considered as an adjustable constant, the total noise in Z consists of two noise components, in which the first one is pure intrinsic noise in Z, the second one is the fluctuation propagated from the neighbor Y (i.e., one-step noise propagation). However, as seen in Equation (35), if we treat X as a variable, then the total noise in Z contains more noise sources, including pure intrinsic noise of Z, one-step propagation noise from X (X regulates Z directly via feed forward loop), one-step propagation noise from neighboring Y, and two-step propagation noise from X (X modulates Z indirectly through Y). In order to study noise propagation in the coherent feed-forward loop, the total noise and different-step noise sources are theoretically discussed in Equations (18) and (35).

**Figure 6:**
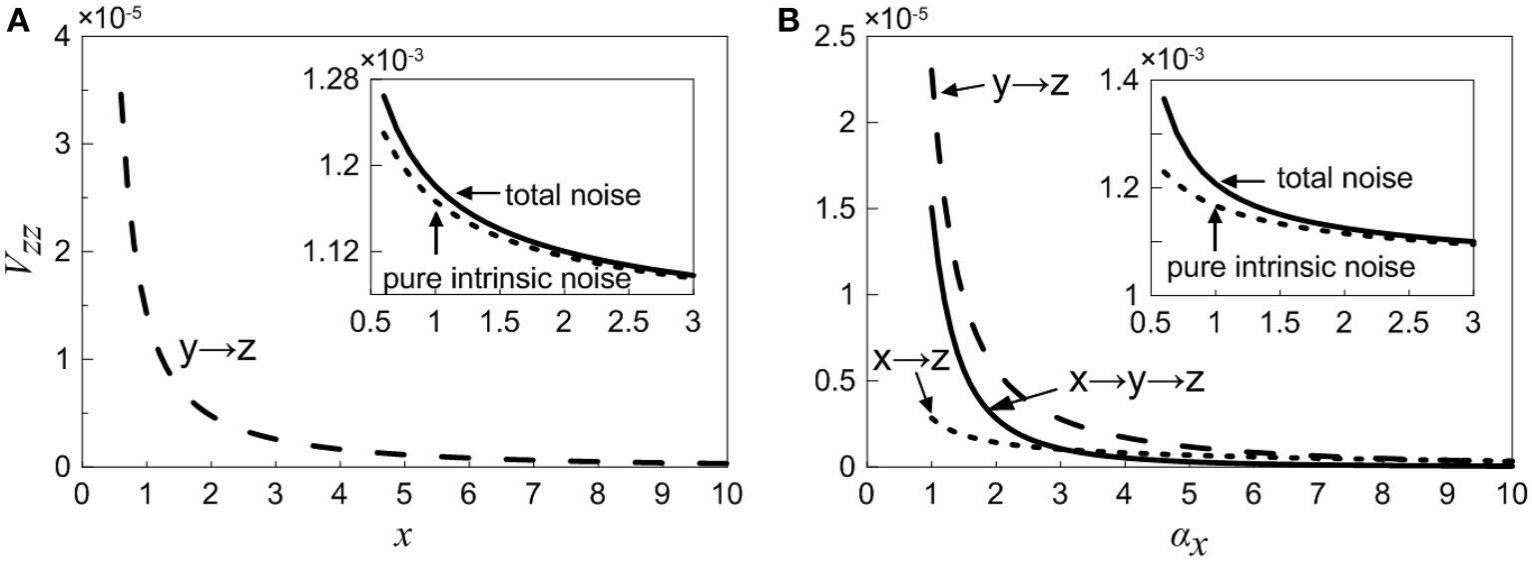
**Noise propagation in coherent feed-forward loop**. **(A)** Treating the concentration of X as an adjustable parameter. **(B)** Treating the concentration of X as a variable. Long point line represents the one-step propagation noise of Y, short dot line represents the one-step propagation noise of X. Solid line shows two-step propagation noise of X. In the inset, solid line shows the total noise and short dot line represents pure intrinsic noise of Z.

Case (i): The concentration of X is an adjustable constant. The inset in Figure 6A shows that the total noise and pure intrinsic noise in Z both vary monotonously with increasing *x*. The noise levels are slightly large at the beginning and then decreases rapidly, which is agreed qualitatively with the fact that molecular noise is negatively correlated with molecular number. Furthermore, it is obvious that the total noise is mainly caused by pure intrinsic noise of Z, and only a small proportion of noise is originated from the one-step propagation. In Figure 6A, the noise term from one-step propagation is plotted. It can be found that with the increase of the concentration of X, the transmitted noise is reduced and finally disappears.
Case (ii): The concentration of X is a variable. The inset in Figure 6B shows that the total noise in Z is also mainly determined by its intrinsic noise. However, the fine structure of noise components can be further observed by our theoretical results. The three curves exhibit clearly the different-steps noise propagation.

### Contributions of upstream and intermediate components to downstream element's total noise

**Figure 7:**
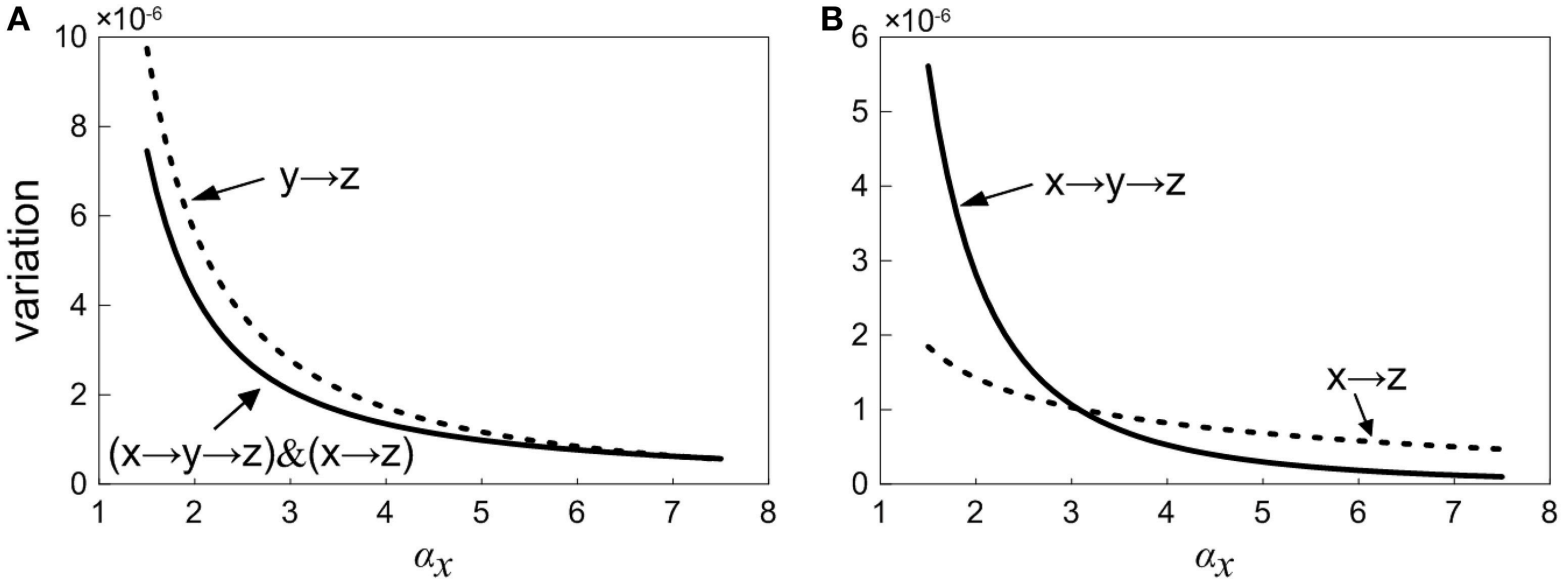
**Normalized variation in Z with increasing the control parameter for different propagation pathway**. **(A)** Solid line shows the noise level including the first-step and two-step noise propagation pathways from X to Z. Short dot line represents the first-step noise propagation pathway from Y to Z. **(B)** Short dot line represents the one-step noise propagation pathway from X to Z. The solid line shows two-step noise propagation pathway from X to Z.

In order to study the propagation feature of the intrinsic expression noise along the direction of signaling transduction, we add the one-step and two-step noise terms of X together. Then, the contribution of noise propagation from X to Z is compared with the contribution from Y to Z, as shown in Figure 7A. It is observed that the noise contribution of the upstream factor X is relatively smaller. With the increasing of the control parameter, the noise contribution to Z from Y becomes close to that from X. Roughly speaking, the noise transmitted from upstream is weaker than the noise propagated from intermediate neighbor.

### Characteristics of noise propagation via main road and branch in the loop

We further consider how the expression noise of upstream component X is transferred to downstream Z via the main road and branch. The curves of noises propagated through main road and branch are plotted in Figure 7B, respectively. It shows that there is an intersection point for two noise curves, i.e., there exists a critical point. When the control parameter is below the critical value, the noise transmitted through the branch is relatively smaller. On the other hand, when the system is beyond the critical value, the noise transmitted via main road is comparatively smaller. It might be caused by the fact that for signaling systems, the regulation of branch occupies the strategic position in the early stage, however, the main road plays a major role in the late stage. Coherent feed-forward loop meets the requirements of different periods. Therefore, our result provides a potential club to explain the reason a large number of coherent feed-forward transcriptional regulatory motifs exists in the cell.

## Conclusion

A population of genetically identical cells exposing to the same extracellular environment may exhibit considerable noise in the mRNA or protein level. This cell-to-cell noise is generated largely due to the limited number of reacting molecules such as gene copies, mRNA, or proteins. Understanding the dynamics of noise propagation in gene regulation systems is an important question on noise analysis in biophysics and system biology.

In this paper, we studied how the expression noise is propagated through a coherent feed-forward loop. First we established a toy yet representative model of gene regulation with feed-forward. Then the theoretical formulas for noise propagation were derived by using the linear noise approximation of master equation and logarithmic gain. We have analytically shown that the total noise is a simple sum of different noise sources including the intrinsic noise and the transmitted noise from other elements in the loop. Therefore, signal transmission is accompanied by noise propagation. In principle, the sub-processes in signal transduction may also contribute to the total noise. By analyzing this decomposition of expression noise, we have further obtained some interesting results about noise characteristics and propagation.

- Compared with other upstream components, the noise level of downstream component is smaller in the coherent feed-forward loop due to the addition of branch. The multi-channel process in the coherent feed-forward loop is advantageous to the propagation of signals, which means that the expression noise level of downstream gene can be reduced due to feed forward loop. Our finding may present a clue to understand why the fate decision system in budding yeast would evolve into a coherent feed forward structure (Li et al., 2015).
- The main noise source in the total noise for downstream component is intrinsic noise. The noise propagated from upstream factor is weaker than the noise transmitted from intermediate component when the system is below this critical point. By comparing the different noise contributions of upstream factors, a transition point in the coherent feed-forward loop is observed. When the system is below this transition point, the noise of the main road is relatively higher while the noise of branch is higher when the system is beyond this point. The two-phase mechanism could be advantageous to the signal propagation.

Phosphotransferase system (PTS) is a signaling network in bacteria, responsible for sensing and using a certain nutrient. In the PTS, enzyme I (EI) is first autophosphorylated and then transfers the phosphoryl group to enzyme IIA-Glucose (EIIA^Glc^) via enzyme histidine phosphorcarrier (HPr). As an alternative pathway, we found that, EIIA^Glc^ can be phosphorylated directly by EI in the absence of HPr. Therefore, the PTS is similar to the above feed-forward system. Recently, using the paramagnetic NMR spectroscopy and mathematical model, we have investigated the mechanism of phosphoryl transfer between the EI and EIIA^Glc^ and demonstrated the physical basis for their ultraweak interaction (Xing et al., 2014). Interestingly, by a further dynamical analysis, a critical point is also found in the PTS system with feed-forward loop. When the concentration of HPr is smaller than a critical value, the main road plays a major role on transfer phosphate, however the transfer of phosphoryl group through the branch is dominated when the system is beyond the point. We hope that the potential relationship between the two transition points in the feed-forward system and the PTS system can be clarified in our next work.

Our research has clarified the potential relationships between feed-forward structure, noise characteristics, and signal transduction. By comparing the properties of three different motifs, we easily find that the feed-forward motif is a best design for signal transduction because it excels in the noise-reduction function. So far, it is the first time to clarify noise characteristics and propagation mechanism in a coherent feed-forward loop by analytical method. The ability to dissect theoretically noise propagation through complex biological networks enables the researchers to understand the role of noise in function and evolution. Our work provides a preliminary result for noise decomposition in gene regulation circuits. However, there still exist some deficiencies in our theoretical work. (i) Because the coherent feed-forward loop has the highest abundance in nature, we have only analyzed this form of coherent feed-forward loop. It is worthy to study the noise transmission in other feed-forward loops. (ii) Due to lack of experimental data, it is difficult to build a quantitative coherent feed-forward loop model. Only a coarse model of coherent feed-forward loop is used to explore the qualitative behaviors. For example, in the reaction process, the Hill coefficient is equal to 1 without taking into account the case of N greater than 1. (iii) We only select one set of parameter values to make a simple analysis. We can see the fine structure of total noise for each component, but the noise level is very small, especially the first-step propagation noise via branch.

## Author contributions

Conceived and designed the experiments: RG, YJ, and MY. Performed the experiments: RG, QL, YY, HD, and CM. Analyzed the data: RG, QL, YY, HD, CM, YJ, and MY. Contributed reagents/materials/analysis tools: RG, QL, YY, HD, and CM. Wrote the paper: RG, YJ, and MY.

### Conflict of interest statement

The authors declare that the research was conducted in the absence of any commercial or financial relationships that could be construed as a potential conflict of interest.
